# Obesity-Induced Inflammation and Insulin Resistance

**DOI:** 10.3389/fendo.2014.00204

**Published:** 2014-12-04

**Authors:** Tsuguhito Ota

**Affiliations:** ^1^Department of Cell Metabolism and Nutrition, Brain/Liver Interface Medicine Research Center, Kanazawa University, Kanazawa, Japan

**Keywords:** obesity, insulin resistance, inflammation, macrophage, innate immunity, adipose tissue, pattern-recognition receptor, bariatric surgery

Immune response and metabolic regulation are highly integrated and this interface maintains a central homeostatic system, dysfunction of which can cause obesity-associated metabolic disorder such as type 2 diabetes, fatty liver disease, and cardiovascular disease. Insulin resistance is an underlying basis for the pathogenesis of these metabolic diseases. Overnutrition or obesity activates the innate immune system with subsequent recruitment of immune cells such as macrophages and T cells, which contributes to the development of insulin resistance. In particular, a significant advance in our understanding of obesity-associated inflammation and insulin resistance has been recognition of the critical role of adipose tissue macrophages (ATMs). ATMs are a prominent source of proinflammatory cytokines, such as TNF-α and IL-6, that can block insulin action in adipose tissue, skeletal muscle, and liver autocrine/paracrine signaling and cause systemic insulin resistance via endocrine signaling, providing a potential link between inflammation and insulin resistance.

All articles in this topic highlight the interconnection between obesity, inflammation, and insulin resistance in all its diversity to the mechanisms of obesity-induced inflammation and role of immune system in the pathogenesis of insulin resistance and diabetes. These articles give some insight into unanswered questions in relation to this topic. First, the relationship between nutrient sensing systems and the interface of metabolic and inflammatory responses is complex. Pattern-recognition receptors (PRRs) such as Toll-like receptors (TLR) and the receptor for advanced glycation end-products (RAGE) are activated in response to dietary nutrients and changes of gut microbiota as described in the review articles by Tanti et al. and Yamamoto et al. (1, 2). The activation of PRRs plays a crucial role as a trigger of this metabolic inflammation. Retinoic acid-related orphan receptors RORα and RORγ, also provide an important link between the circadian clock machinery and its regulation of metabolic genes (3). Two studies by Moustaid-Moussa et al. show a potential role of the renin angiotensin system in the development of insulin resistance and diabetes by using gain and loss of function in mice (4, 5). Second, it is not clear which cell recruits and activates or which tissue inflammation initially occurs upon obesity, which then causes systemic inflammation and subsequent development of insulin resistance. McArdle et al. and Tateya et al. describe how obesity causes alteration of the immune cells, in which TH1 cells, B cells, neutrophils, or mast cells induce M1 activation and polarization of macrophages by the elevated secretion of TNFα and IFNγ (6, 7). In addition to proinflammatory cytokines and adipokines, more metabolic regulators including fibroblast growth factor (FGF) family such as FGF 21 and FGF19 and bioactive lipids, sphingolipids can contribute to the development of systemic inflammation and subsequent development of insulin resistance (8, 9). Interestingly, these novel adipokines can be dramatically changed after bariatric surgery and these changes contribute to improvement of obesity-associated inflammation, insulin resistance, and glucose homeostasis (10).

Overall, all the original articles and review articles covering this topic in all its diversity to contribute somewhat to clarify unanswered questions on the mechanisms of obesity-induced inflammation and insulin resistance.

## Conflict of Interest Statement

The author declares that the research was conducted in the absence of any commercial or financial relationships that could be construed as a potential conflict of interest.
